# Structure-Dependent Effects of Bisphosphonates on Inflammatory Responses in Cultured Neonatal Mouse Calvaria

**DOI:** 10.3390/antiox9060503

**Published:** 2020-06-09

**Authors:** Keiko Suzuki, Sadaaki Takeyama, Shinobu Murakami, Masahiro Nagaoka, Mirei Chiba, Kaoru Igarashi, Hisashi Shinoda

**Affiliations:** 1 Department of Dental Pharmacology, School of Dentistry, Showa University, Tokyo 142-8555, Japan; 2 Department of Molecular Cell Pharmacology, Graduate School of Dental Medicine, Hokkaido University, Sapporo 060-8586, Japan; sadaakitake@gmail.com; 3 Center for Environmental Dentistry, Graduate School of Dentistry, Tohoku University, Sendai 980-8575, Japan; shi.mura531@gmail.com (S.M.); hisashi.shinoda.b4@tohoku.ac.jp (H.S.); 4 Department of Dental Pharmacology, School of Dentistry, Ohu University, Fukushima 963-8611, Japan; m-nagaoka@den.ohu-u.ac.jp; 5 Division of Oral Physiology, Tohoku University Graduate School of Dentistry, Sendai 980-8575, Japan; mirei.chiba.d6@tohoku.ac.jp; 6 Division of Craniofacial Anomalies, Tohoku University Graduate School of Dentistry, Sendai 980-8575, Japan; igarashi@tohoku.ac.jp

**Keywords:** bisphosphonate, antioxidant, anti-inflammatory, PGE_2_, NO, peroxynitrite, NF-κB, nitrotyrosine, bisphosphonate-related osteonecrosis of the jaw (BRONJ), periodontitis

## Abstract

Bisphosphonates (BPs) are classified into two groups, according to their side chain structures, as nitrogen-containing BPs (NBPs) and non-nitrogen-containing BPs (non-NBPs). In this study, we examined the effects of NBPs and non-NBPs on inflammatory responses, by quantifying the inflammatory mediators, prostaglandin E_2_ (PGE_2_) and nitric oxide (NO), in cultured neonatal mouse calvaria. All examined NBPs (pamidronate, alendronate, incadronate, risedronate, zoledronate) stimulated lipopolysaccharide (LPS)-induced PGE_2_ and NO production by upregulating *COX-2* and *iNOS* mRNA expression, whereas non-NBPs (etidronate, clodronate, tiludronate) suppressed PGE_2_ and NO production, by downregulating gene expression. Additionally, [4-(methylthio) phenylthio] methane bisphosphonate (MPMBP), a novel non-NBP with an antioxidant methylthio phenylthio group in its side chain, exhibited the most potent anti-inflammatory activity among non-NBPs. Furthermore, results of immunohistochemistry showed that the nuclear translocation of NF-κB/p65 and tyrosine nitration of cytoplasmic protein were stimulated by zoledronate, while MPMBP inhibited these phenomena, by acting as a superoxide anion (O_2_^−^) scavenger. These findings indicate that MPMBP can act as an efficacious agent that causes fewer adverse effects in patients with inflammatory bone diseases, including periodontitis and rheumatoid arthritis.

## 1. Introduction

Bisphosphonates (BPs) are well recognized as potent inhibitors of osteoclastic bone resorption and have been successfully used for the treatment of various metabolic bone diseases, such as osteoporosis, Paget’s disease, hypercalcemia, and osteolytic tumor-induced bone diseases, which cause excessive bone resorption [1]. All BPs are chemically characterized by a P-C-P bond, and therefore, share common properties (i.e., a strong binding affinity for hydroxyapatite in bone tissue [2] and potent anti-resorption activity [3]). However, their pharmacological characteristics, including potency of anti-resorption activity, mechanism of action, and adverse effects vary considerably, depending on their side chain structures. BPs are categorized into two groups, based on whether the side chains contain a nitrogen atom, as nitrogen-containing BPs (NBPs) and non-nitrogen-containing BPs (non-NBPs). Furthermore, it is well known that this classification corresponds well to differences in the mechanism of action and the incidence of adverse effects. With regard to osteoclast inactivation, NBPs have been reported to inhibit farnesyl pyrophosphate synthase in the mevalonate pathway in osteoclasts [4], thereby preventing the prenylation of small G proteins required for osteoclast function, while non-NBPs form cytotoxic metabolites that compete with ATP in energy metabolism. Both of these can cause apoptosis in osteoclasts [5]. Among the adverse effects of BPs, the incidence of bisphosphonate-related osteonecrosis of the jaw (BRONJ), characterized by an exposed necrotic jaw bone [6] filled with bacterial aggregates, has been reported to be much higher in patients receiving long-term and/or high-dose intravenous NBP administration as compared to those administered non-NBPs [7,8,9]. Although the reasons for the difference between patients receiving NBPs and non-NBPs have remained uncertain, there are some possible explanations: (i) NBPs are prone to cause hyperinflammation, which induces oral mucosal tissue injury [10] and (ii) NBPs strongly suppress the bone turnover due to their potent anti-osteoclastic activity, which results in delayed bone healing, as compared to that in patients receiving non-NBPs [11].

The novel compound [4-(methylthio) phenylthio] methane bisphosphonate (MPMBP) is a non-NBP with an antioxidative methylthio phenylthio group in its side chain [12]. In previous studies focusing on the structure–activity relationships among various BPs, we found that MPMBP (research code number: TRK-530) possessed several unique functions that could not be observed in NBPs, such as (i) inhibition of superoxide anion (O_2_^−^) generation in human polymorphonuclear leukocytes (PMN) [13], (ii) prevention against mouse collagen-induced arthritis [14] and rat adjuvant arthritis [15,16], characterized by persistent inflammation in the joints [17], and (iii) prevention against inflammatory alveolar bone loss in experimental periodontitis [18]. We also observed that there were several features that were common between BPs, including the inhibition of bone resorption, both in organ culture [18] and in the animal model of inflammatory osteolysis [19]. Since an excessive and chronic inflammatory response can damage the host cells and tissues [20], which in turn can be the risk factor for BRONJ, the anti-inflammatory activity resulting from the anti-oxidative property of MPMBP might lower the incidence of adverse effects including BRONJ.

In the present study, we examined the effects of NBPs and non-NBPs on lipopolysaccharide (LPS)-induced inflammatory responses, by measuring the levels of inflammatory mediators, prostaglandin E_2_ (PGE_2_), and nitric oxide (NO) [21], in the cultured neonatal mouse calvaria. Additionally, we investigated the possible mechanisms through which MPMBP suppressed inflammatory responses in the mouse calvaria, which contain immune cells as well as bone cells.

## 2. Materials and Methods

### 2.1. Calvaria Culture

Organ culture of neonatal mouse calvaria has been described in detail previously [22]. In brief, calvaria were dissected from 4–6-day-old ddY mice bred in the Animal Facility of Tohoku University (originally from Japan SLC Co., Shizuoka, Japan) and cultured free-floating in glass roller tubes in Dulbecco’s modified Eagle medium (DMEM; Gibco) supplemented with 10% heat-inactivated fetal calf serum and antibiotics. After 24 h of pre-culture period, media was changed to fresh media with or without lipopolysaccharide (LPS; O111 from *Escherichia coli*; Wako Pure Chemical Industries, Ltd., Osaka, Japan) (either 1 or 10 μg/mL according to the manufacturer’s specification for the production lots), and cultured for a further 24 or 48 h in the presence of NBPs (pamidronate, alendronate, incadronate, risedronate or zoledronate) or non-NBPs, (etidronate, clodronate, tiludronate, or disodium dihydrogen-4-(methylthio) phenylthio methane bisphosphonate (MPMBP), a newly developed BP). Etidronate, clodronate, incadronate, and risedronate were synthesized and supplied by Proctor and Gamble Pharmaceutics’ Wood Corners Laboratories (Cincinnati, OH, USA). Alendronate and pamidronate were purchased from Sigma-Aldrich Co. (St. Louis, MO, USA) and zoledronate was purchased from Novartis Pharma AG (Tokyo, Japan). Tiludronate and MPMBP were synthesized and supplied by Basic Research Laboratories at Toray Industries Inc., (Kamakura, Japan). NOC18 (1-hydroxy-2-oxo-3, 3-bis (2-aminoethyl)-1-thiazene) and SIN-1 (3-(4-morpholinyl) sydnonimine hydrochloride) were purchased from the Dojindo-Lab (Kumamoto, Japan). The animals were treated ethically in compliance with the commonly accepted ‘3Rs‘—Replacement, Reduction, Refinement—in accordance with the protocol, which was approved by the “Regulations for animal experiments and related activities at Tohoku University (2008DnA-33)” and “Showa University Animal Care and Use Committee guidelines (#10076).”

### 2.2. Effects of NBPs and Non-NBPs on PGE_2_ and NO Production in Calvaria

The effects of BPs (1–125 μM for etidronate, clodronate, and MPMBP or 0.1–62.5 μM for pamidronate, alendronate, risedronate and incadronate, or 0.2–25 μM for zoledronate) on the inflammatory responses in the calvaria were assessed by measuring PGE_2_ and NO levels in culture media harvested at 24 h (preculture), 48 h (0–24 h), and 72 h (0–48 h) after treatment with BPs. The concentration of PGE_2_ was determined using the enzyme immunoassay kit (EIA; Amersham Pharmacia Biotech UK Ltd., Buckinghamshire, UK). NO production was determined by measuring the end products of NO (i.e., nitrite (NO_2_^−^) and nitrate (NO_3_^−^)), in the culture media by the Griess method, using an automated NO detector-HPLC system (ENO-10, Elcom, Kyoto, Japan). In brief, NO_2_^−^ and NO_3_^−^ released into the media, were separated by a reverse-phase separation column packed with polystyrene polymer, and then, NO_3_^−^ was reduced to NO_2_^−^ using a reduction column packed with copper-plated cadmium fillings. Samples containing NO_2_^−^ were mixed with a Griess reagent, which resulted in the diazotization of sulfanilamide by NO_2_^−^ and its subsequent coupling to N-(1-naphthyl) ethylene diamine, to form an azo dye, which was detected by measuring the absorbance at 540 nm.

### 2.3. Reverse Transcription-PCR (RT-PCR) Analyses of Cultured Calvaria

Total RNA was isolated from cultured calvaria using TRIzol (Invitrogen Ltd., Waltham, MA, USA). The cDNA was synthesized from 5 μg of total RNA using the SuperScript^TM^ First-Strand Synthesis System for RT-PCR (Invitrogen Ltd., Waltham, MA, USA). RT-PCR was performed using a thermal cycler (iCycler, Bio-Rad Laboratories Inc., Tokyo, Japan). Quantitative RT-PCR (qPCR) was performed in a Thermal Cycler Dice Real Time System (TP-870; Takara Bio Inc., Shiga, Japan), using SYBR Premix Ex Taq Perfect Real Time (Takara Bio Inc., Shiga, Japan), according to the manufacturer’s protocol. The sequence of the specific primers for the qPCR is listed in Table 1. The level of mRNA expression was normalized with that of β-actin.

### 2.4. Fluorescent Staining of Calvaria and Laser Scanning Confocal Microscopy

Fluorescent staining of cultured calvaria was performed as described previously [22]. Briefly, calvaria were cut into four pieces along the sutures and only two pieces of parietal bones were used in this experiment. Bone pieces were washed with phosphate-buffered saline (PBS), blocked with 2% bovine serum albumin (BSA)–0.01% Triton X-100 in PBS (BSA buffer), and incubated with primary antibodies (diluted 1:100 in BSA buffer) overnight at 4 °C, followed by appropriate secondary antibodies, either Alexa Fluor 488^TM^- or 594^TM^-labeled antibodies (diluted 1:200 in BSA buffer; Invitrogen Co., Carlsbad, CA, USA), for 2 h. Goat anti-NF-κB/p65 antibody (C-20, sc-372-G), rabbit polyclonal iNOS antibody, and mouse anti-nitrotyrosine antibody (7A12AF6, ab110282; recognizes only protein-bound nitrotyrosine) were from Santa Cruz Biotechnology, Inc. (Dallas, TX, USA), Novus Biologicals, (Centennial, CO, USA) and Abcam (Cambridge, UK), respectively. Alkaline phosphatase (ALP) activity was detected using an ELF 97 endogenous phosphatase detection kit (Molecular Probes, Eugene, OR, USA). Then, 6-diamidino-2-phenylindole (DAPI) was used for nuclear counterstaining. After fluorescent staining, the calvaria were examined using a laser scanning confocal microscope (FV1000; Olympus Optical Co., Tokyo, Japan), as described previously [22]. The confocal images were obtained using FV 10-ASW 4.2, an image-processing and analysis software (Olympus Optical Co.).

### 2.5. Western Blot Analysis in Cultured Calvaria

Cultured calvaria were lysed with lysis buffer (50 mM Tris-HCl, 1% Nonidet^TM^ P-40, 0.25% sodium deoxycholate, 150 mM NaCl, 1 mM EDTA, and 1 mM PMSF), after performing sonication (15 s). The sample solution (50 μg of protein) was incubated with the anti-COX-2 antibody (Santa Cruz Biotechnology Inc.,) for 3 h and with protein G-agarose for 2 h. Immunoprecipitates were washed several times with PBS, separated by 10% sodium dodecyl sulfate polyamide gel electrophoresis (SDS-PAGE), and transferred onto PVDF membranes (Immobilon Transfer Membrane, Millipore Co., Bedford, MA, USA). After blocking the membrane with 5% skimmed milk, it was incubated with the anti-COX-2 antibody (1:1000; Santa Cruz Biotechnology Inc.) for 3 h, followed by incubation with horseradish peroxidase-coupled anti-goat IgG (1:3000; Santa Cruz Biotechnology Inc.) for 1 h. Immunoreactive protein bands were detected using the ECL chemiluminescence detection kit (Amersham Pharmacia Biotech, Buckinghamshire, UK).

### 2.6. Effects of Non-NBPs on the Generation of Superoxide Anions in the Xanthine-Xanthine Oxidase System 

2-Methyl-6-phenyl-3,7-dihydroimidazo [1,2-a] pyrazin-3-one, a Cypridina luciferin analogue and a chemiluminescent probe (CLA, Tokyo Kasei Kogyo Co. Ltd., Tokyo, Japan) was dissolved in ultrapure water and stored at −20 °C until use. Diethylentriamine-N,N,N′,N″,N″-pentaacetic acid (DTPA; Wako Pure Chemical Industries Ltd., Osaka, Japan) and various BPs were dissolved in Hanks’ balanced buffered saline solution (HBSS; Gibco). The reaction mixture containing 1 µM CLA, 100 µM DTPA, 100 µM xanthine (Sigma Chemical Co., Saint Louis, MO, USA), and BPs was pre-incubated for 2 min at 37 °C. Then the reaction was started with the addition of 0.5 U/mL xanthine oxidase (Roche Diagnostics Co., Basel, Switzerland) to the reaction mixture at 37 °C. The amount of superoxide anion (O_2_^−^) was determined by measuring the integral amount of light emission at 380 nm for 10 s with a luminometer (Luminescencer; Atto Bioscience and Biotechnology, Tokyo, Japan).

### 2.7. Statistical Analysis

Statistical analyses were performed using one-way analysis of variance (ANOVA) for comparison among all groups. The Tukey’s honestly significant difference (HSD) test was performed for post hoc pair-wise comparisons after the ANOVA with software KaleidaGraph (HULINKS Inc., Tokyo, Japan). The data were from a minimum of four values in replicate experiments. A *p*-value less than 0.05 was considered statistically significant and only the significance level compared with the LPS-alone or control group was depicted, as indicated in the figure legends.

## 3. Results

### 3.1. Effects of NBPs and Non-NBPs on PGE_2_ Production in Cultured Calvaria

**Figure 1:**
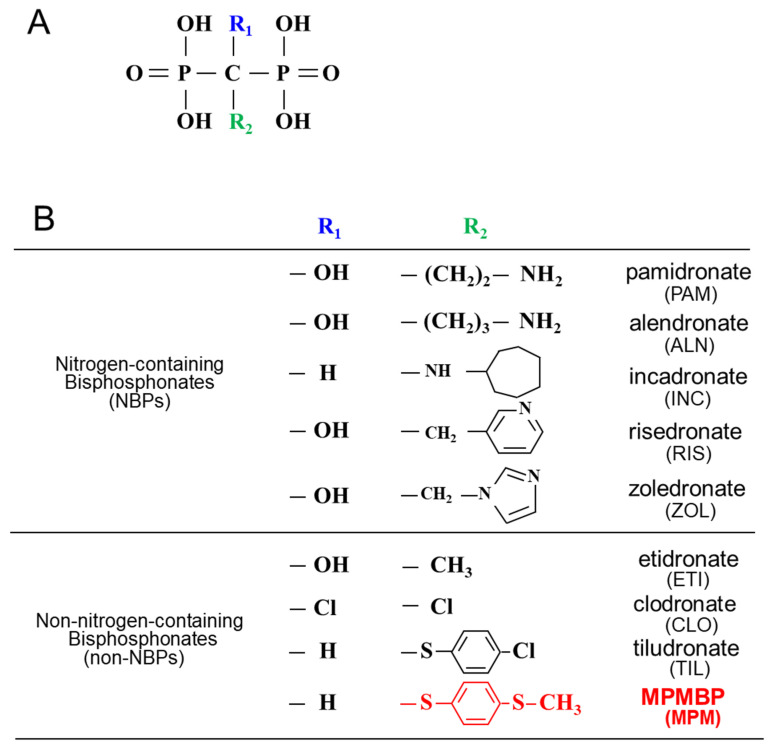
Chemical structure of bisphosphonates. (**A**) General formula of bisphosphonates; (**B**) chemical structure of nitrogen-containing bisphosphonates (NBPs) and non-NBPs used in the present investigation. Note that [4-(methylthio) phenylthio] methane bisphosphonate (MPMBP), a novel non-NBP, possesses an antioxidant side chain (R2; -S-phenyl-S-CH_3_) attached to the central carbon atom.

**Figure 2:**
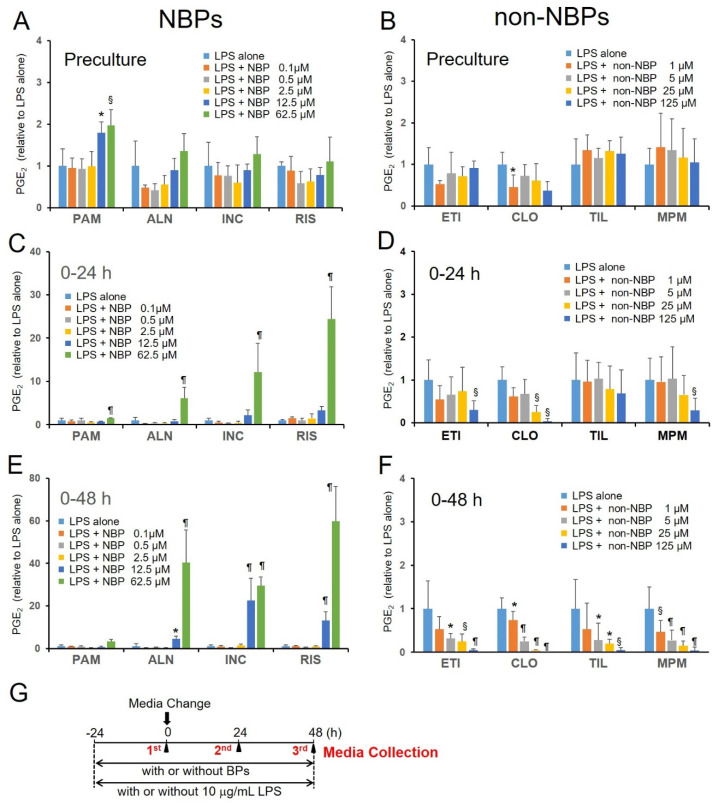
Effects of various BPs on lipopolysaccharide (LPS)-induced prostaglandin E_2_ (PGE_2_) production in cultured neonatal mouse calvaria. (**A**–**F**) The amount of PGE_2_ that was produced and secreted into the media during the indicated time periods was measured by EIA. Calvaria were cultured free-floating in roller tubes with or without NBPs (0.1–62.5 μM; **A**,**C**,**E**) and non-NBPs (1–125 μM; **B**,**D**,**F**), in the presence of 10 μg/mL LPS. (**G**) Experimental protocol for organ culture of neonatal mouse calvaria. Values are expressed as mean ± SD (*n* = 5–12). *: *p* < 0.05, §: *p* < 0.01, ¶: *p* < 0.001 versus LPS-alone.

Since one of the major causes of BRONJ is persistent inflammation induced by host defense against bacterial infections in the oral cavity, we first explored whether NBPs and non-NBPs have differential effects on LPS-induced inflammatory responses, by measuring PGE_2_ production. Calvaria from neonatal mice were pre-incubated in the presence of various NBPs (pamidronate, alendronate, incadronate, and risedronate; Figure 1B) or non-NBPs (etidronate, clodronate, tiludronate, and MPMBP; Figure 1B) for 24 h, to exclude the PGE_2_ produced by mechanical stimulation, during the calvaria dissection process (Figure 2A,B).

After changing the media to fresh media containing individual BPs, the calvaria were incubated for another 24 h (Figure 2C,D) or 48 h (Figure 2E,F). During the culture period, any toxic effects of BPs were observed under a microscope within the range of 0.1–62.5 μM of pamidronate, alendronate, incadronate, and risedronate, 0.2–25 μM of zoledronate, and 1–125 μM of etidronate, clodronate, tiludronate, and MPMBP (Figure S1). Since the PGE_2_ production levels in the LPS-alone group were not significantly different across either different culture periods or different BP treatments, values were expressed as the ratio over the average value for the LPS-alone group in each experiment. As shown in Figure 2C,E, all the NBPs tested stimulated the LPS-induced PGE_2_ production in a time- and dose-dependent manner, indicating that NBPs might enhance inflammatory responses through a mechanism involving accelerated PGE_2_ production. On the contrary, non-NBPs inhibited LPS-induced PGE_2_ production in a dose-dependent manner within the 48 h-long culture period (Figure 2D,F).

**Figure 3:**
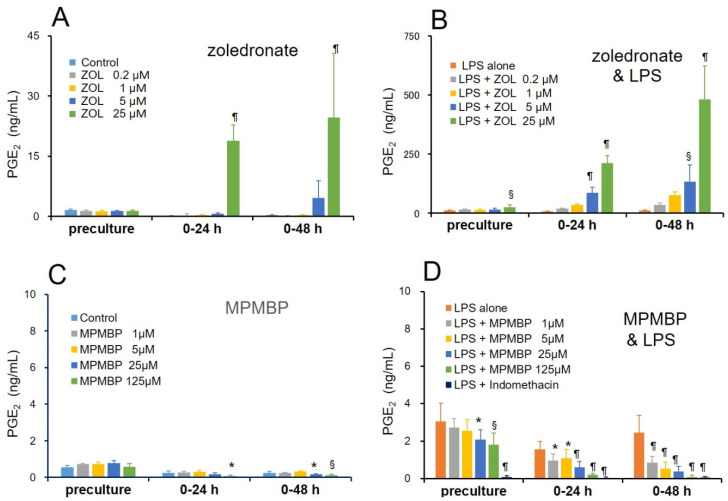
Effects of an NBP zoledronate (0.2–25 μM) and a non-NBP MPMBP (1–125 μM) on the basal and LPS-induced PGE_2_ production in cultured neonatal mouse calvaria. (**A**–**D**) The amount of PGE_2_ produced and secreted into the media during the indicated time periods was measured by EIA. Calvaria were cultured free-floating in roller tubes with or without zoledronate (**A**,**B**) and MPMBP (**C**,**D**) in the absence (**A**,**C**) or presence (**B**,**D**) of 10 μg/mL LPS. Values are expressed as mean ± SD (*n* = 5–7). *: *p* < 0.05, §: *p* < 0.01, ¶: *p* < 0.001 versus either control (**A**,**C**) or LPS-alone (**B**,**D**).

Next, we investigated the differential effects of zoledronate, the NBP known to exhibit the most potent antiresorptive activity and cause BRONJ most frequently, and MPMBP, a newly developed non-NBP with an antioxidant side chain, on PGE_2_ production in the same experimental settings. As expected, 25 μM of zoledronate significantly stimulated the basal level of PGE_2_ production by 90- and 83-fold after culturing for 24 h and 48 h, respectively, as compared to that observed for the control (Figure 3A). Furthermore, in the concomitant presence of 10 μg/mL LPS, 5 μM and 25 μM of zoledronate stimulated PGE_2_ production 165- and 402-fold at 24 h, and 384- and 1383-fold at 48 h of culture, respectively, as compared to that of the control (Figure 3B). In contrast, 1–125 μM of MPMBP significantly inhibited LPS-induced PGE_2_ production in a time- and dose-dependent manner (Figure 3D). On the other hand, indomethacin, a nonselective COX-1 and COX-2 inhibitor, completely inhibited LPS-induced PGE_2_ production before the end of the pre-culture period (Figure 3D). The inhibitory effect of MPMBP on PGE_2_ production, however, reached a maximum at 48 h after the pre-culture, indicating that MPMBP did not directly inhibit the activity of the PGE_2_ synthesizing enzyme.

### 3.2. Effects of NBPs and Non-NBPs on COX-2 Expression in Cultured Calvaria

**Figure 4:**
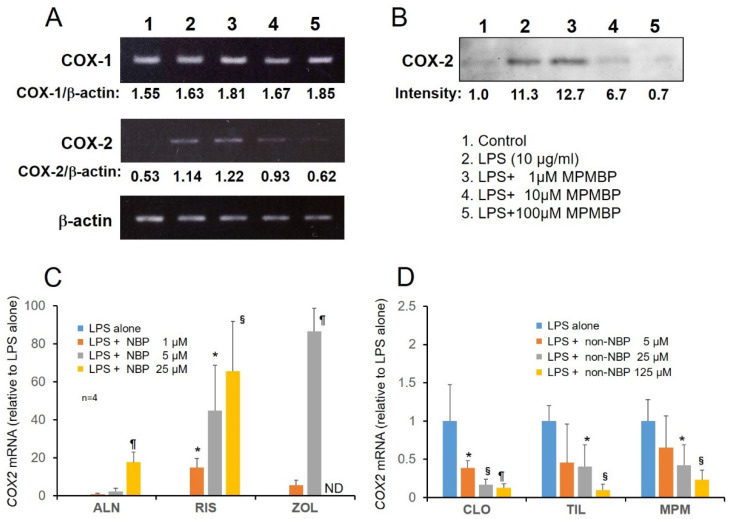
Analyses of gene expression levels in cultured calvaria. (**A**) RT-PCR analysis of two cyclooxygenase isozymes *COX-1* and *COX-2.* The mRNA expression in the calvaria treated with MPMBP (1–100 μM). (**B**) Western blot analysis of COX-2 protein expression in the calvaria treated with MPMBP (1–100 μM). (**C**,**D**) qPCR analysis of the expression of *COX-2*, inducible cyclooxygenase, after incubation with various NBPs (1–25 μM) and non-NBPs (5–125 μM) for 48 h. Values are expressed as mean ± SD (*n* = 4). *: *p* < 0.05, §: *p* < 0.01, ¶: *p* < 0.001 versus LPS-alone. ND: not determined.

As shown in Figure 4A,B (middle panel), RT-PCR and Western blotting analyses showed that 1–100 μM MPMBP suppressed inducible COX-2 expression at both the mRNA and protein levels in cultured calvaria, whereas the constitutive *COX-1* mRNA remained unaffected by MPMBP (Figure 4A, upper panel). The results of qPCR analyses showed that inducible *COX-2* mRNA was upregulated by NBPs (alendronate, risedronate, and zoledronate; Figure 4C) and downregulated by non-NBPs (clodronate, tiludronate, and MPMBP; Figure 4D), in the presence of 10 μg/mL of LPS in the cultured calvaria.

### 3.3. Effects of NBPs and Non-NBPs on the Production of NO and Expression of iNOS mRNA in Cultured Calvaria

**Figure 5:**
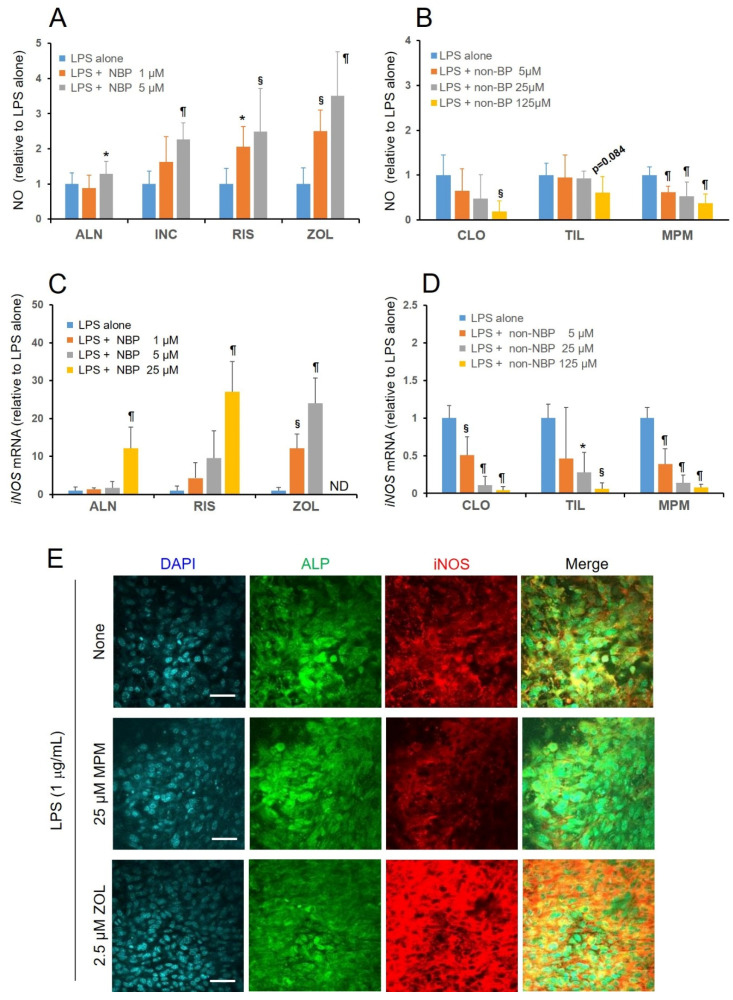
Effects of NBPs and non-NBPs on the nitric oxide (NO) production and *iNOS* expression in neonatal mouse calvaria. (**A**,**B**) The amount of NO produced by calvaria was measured as the end products (nitrite and nitrate) by the Griess method, using an automated NO detector-HPLC system. Calvaria were cultured free-floating in glass roller tubes with or without NBPs (**A**) and non-NBPs (**B**) for 48 h in the presence of 10 μg/mL LPS. Values are expressed as mean ± SD (*n* = 8). *: *p* < 0.05, §: *p* < 0.01, ¶: *p* < 0.001 versus LPS-alone. (**C**,**D**) The qPCR analysis of the expression of *iNOS,* after incubating with various NBPs (**C**) and non-NBPs (**D**) for 48 h, in the presence of 10 μg/mL LPS. Values are expressed as mean ± SD (*n* = 4). *: *p* < 0.05, §: *p* < 0.01, ¶: *p* < 0.001 versus LPS-alone. (**E**) Representative confocal microscopy images of fluorescent-stained calvaria after culturing them with MPMBP or zoledronate for 48 h, in the presence of 1 μg/mL LPS. Scale bars = 40 μm.

To further elucidate the differential effects on inflammatory responses between NBPs and non-NBPs, we investigated the effects of NBPs and non-NBPs on LPS-induced NO production and *iNOS* mRNA expression, under the same experimental conditions as those of PGE_2_ production and *COX-2* mRNA expression. A significant increase in the LPS-induced NO production (Figure 5A), and a concomitant increase in *iNOS* expression (Figure 5C), was observed in all the examined NBPs. On the contrary, MPMBP (5–125 μM) significantly suppressed LPS-induced NO production (Figure 5B), with concomitant downregulation of *iNOS* mRNA (Figure 5D), whereas *nNOS* and *eNOS* mRNA expression levels were assessed to be negligible (data not shown), suggesting that MPMBP prevented LPS-induced excessive inflammation. Moreover, tiludronate failed to inhibit LPS-induced NO production significantly at either tested concentration, even though *iNOS* mRNA expression was downregulated. Immunohistochemical analysis also showed that iNOS protein expression was suppressed by MPMBP (Figure 5E, middle panel) and enhanced by zoledronate (Figure 5E, lower panel), in the calvaria cultured under the same conditions as those of NO production and *iNOS* mRNA expression.

### 3.4. Signaling Pathway through which MPMBP Inhibits the Production of Inflammatory Mediators

**Figure 6:**
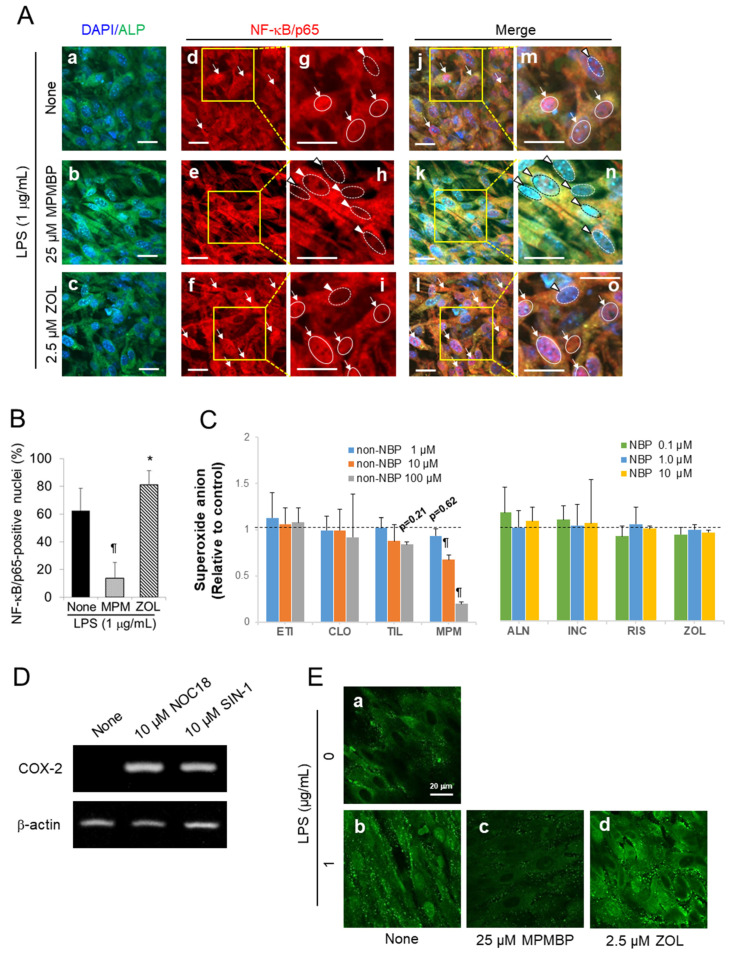
Signaling pathway through which MPMBP inhibits the production of inflammatory mediators. (**A**) Fluorescent staining of cultured neonatal mouse calvaria for alkaline phosphatase activity and immunofluorescent staining for NF-κB/p65. Parietal bones were incubated with the vehicle (**a**,**d**,**g**,**j**,**m**), 25 μM MPMBP (**b**,**e**,**h**,**k**,**n**) or 2.5 μM zoledronate (**c**,**f**,**i**,**l**,**o**) for 48 h and then stimulated with LPS for 4 h. Scale bars = 20 μm. Arrows indicate the nuclear translocation of NF-κB/p65 (solid line circles). Arrowheads indicate the lack of NF-κB/p65 staining in the nuclei (dotted line circles). Figures in the 3rd (**g**–**i**) and 5th (**m**–**o**) columns correspond to the squares in the 2nd (**d**–**f**) and 4th (**j**–**l**) columns, respectively. (**B**) Percentage of NF-κB/p65-positive nuclei in the selected region of interest (ROI)s that contained a relevant number of cells (ranged from 25 to 68 cells) of the parietal bones stained for NF-κB/p65 (mean ± SD, sample size of *n* = 7 per group, *: *p* < 0.05, ¶: *p* < 0.001 versus LPS-alone). (**C**) Effects of non-NBPs (**left panel**) and NBPs (**right panel**) on O_2_^−^ generation in the xanthine-xanthine oxidase system. (mean ± SD, *n* = 5. *: *p* < 0.05, ¶: *p* < 0.001 versus control). (**D**) RT-PCR analysis of the expression of *COX-2* in calvaria cultured with 10 μM NOC18, an NO donor, or 10 μM SIN-1, an ONOO^−^ donor, for 72 h. (**E**) Representative confocal microscopy images of cultured neonatal mouse calvaria immunofluorescent stained for nitrotyrosine (**a**–**d**). Parietal bones were treated with the vehicle (**a**,**b**), 25 μM MPMBP (**c**), or 2.5 μM zoledronate (**d**) for 48 h, and then incubated for a further 4 h in the absence (**a**) or presence of LPS (**b**,**c**,**d**). Scale bars = 20 μm.

LPS is known to activate the transcription factors NF-κB and activator protein 1 (AP-1) through its binding to Toll-like receptor 2 or 4 (TLR2/4), and consequently upregulates a large number of genes, including *COX-2* and *iNOS,* involved in inflammatory responses, in many cell types. Of these transcription factors, NF-κB has been reported to be essential for chronic inflammation [23], which is a hallmark feature of the BRONJ. To explore whether BPs affect the translocation of NF-κB to the nuclei, we analyzed the cultured calvaria by laser scanning confocal microscopy. Immunostaining for NF-κB/p65, an active subunit of NF-κB, revealed that 25.0 μM MPMBP inhibited the LPS-induced nuclear translocation of p65, which was clearly recognized as cytoplasmic staining for p65 (red, arrowheads in Figure 6Ah), and only nuclear staining with DAPI (blue) was observed in the merged image, in MPMBP-treated calvaria (arrowheads in Figure 6An). On the contrary, many nuclei appeared to be pink colored, as a result of the blue color of DAPI and red color of Alexa 594-labeled p65 in the merged images in ALPase-positive (green) cells, which were likely to be osteoblasts, in the LPS-alone (arrows in Figure 6Am), and LPS and zoledronate (arrows in Figure 6Ao) groups. Taken together, these results indicate that NF-κB inactivation in LPS-stimulated calvaria was one of possible mechanisms through which MPMBP prevented the inflammatory response.

To further investigate the mechanism by which MPMBP exerts suppressive effects on the inflammation process, we measured the levels of superoxide anion (O_2_^−^) generated in the xanthine-xanthine oxidase system, because NF-κB is known to be an oxidative stress-responsive transcription factor. As shown in Figure 6C (left panel), out of all the tested non-NBPs, 10 μM and 100 μM of MPMBP significantly inhibited the production of O_2_^−^, while either etidronate or clodronate did not affect O_2_^−^ production. Furthermore, none of the NBPs influenced O_2_^−^ production (Figure 6C, right panel). To elucidate the contribution of peroxynitrite (ONOO^−^), an extremely reactive molecule formed from O_2_^−^ and NO [24] to *COX-2* expression, which leads to the excessive production of PGE_2_. Neonatal mouse calvaria were cultured with 10 μM of NOC18, an NO-releasing compound, or 10 μM SIN-1, an ONOO^−^ donor that continuously liberates O_2_^−^ and NO for 72 h. RT-PCR analysis of the samples obtained from the cultured calvaria showed that both NOC18 and SIN-1 stimulated *COX-2* expression (Figure 6D), indicating the involvement of ONOO^−^ in the excessive production of PGE_2_, through the upregulation of the *COX-2* gene. Additionally, confocal images of calvaria stained with the anti-nitrotyrosine antibody after they were cultured with BPs revealed that 25 μM MPMBP (Figure 6Ec) suppressed LPS-induced tyrosine nitration, while 2.5 μM zoledronate (Figure 6Ed) enhanced this phenomenon.

## 4. Discussion

The organ culture of neonatal mouse calvaria has some advantages over the single cell culture or co-culture systems. In the organ culture, both the cytoarchitecture of individual cells, including immune cells (neutrophils, lymphocytes, and macrophages), along with bone cells and the intercellular connections, are thought to be well retained within their natural environment. In addition, they are not influenced by systemic factors. By using this culture system, we investigated the association between the nitrogen-containing side chain of BPs and hyperinflammation levels, as measured by the production of PGE_2_ and NO. The results showed that all the examined NBPs (pamidronate, alendronate, incadronate, risedronate, and zoledronate) enhanced LPS-induced inflammatory responses, while all non-NBPs (etidronate, clodronate, tiludronate, and MPMBP) suppressed inflammatory mediator production. Since the results of crystal structure analysis by Hosfield showed that the existence of the nitrogen atom in NBPs was essential for the inhibition of farnesyl pyrophosphate (FPP) synthase [25], a key enzyme in the mevalonate pathway, the mechanism for stimulating inflammation is thought to be identical among NBPs. On the other hand, the mechanisms explaining the anti-inflammatory effects of non-NBPs are likely to be different from each other, because non-NBPs do not share the common chemical structures in their side chains. It is generally accepted that non-NBPs act as cytotoxic ATP analogues in the osteoclasts; however, there is no evidence that MPMBP forms an ATP analogue, through which it inhibits osteoclastic bone resorption or other functions.

In the present study, we demonstrated that MPMBP exhibited the most potent anti-inflammatory properties against both the basal and LPS-induced production of PGE_2_ and NO, by downregulating the expression of *COX-2* and *iNOS* mRNA. Since NF-κB is well known to be a crucial transcription factor for inflammation-related genes, including *COX-2* and *iNOS*, we observed the cultured calvaria by using confocal microscopy, in order to examine the contribution of NF-κB to the excessive production of PGE_2_ and NO that leads to hyperinflammation. The results showed that the nuclei of an osteoblastic marker for ALP-positive cells were stained positive for NF-κB/p65 after LPS stimulation, indicating that NF-κB was activated in the osteoblasts in response to LPS. Under the same culture conditions, zoledronate stimulated the nuclear translocation of NF-κB/p65 in the calvaria, while MPMBP inhibited the nuclear translocation of NF-κB/p65, as evidenced by the cytoplasmic staining for inactive form of NF-κB/p65 in ALP-positive cells (Figure 6A).

**Scheme 1:**
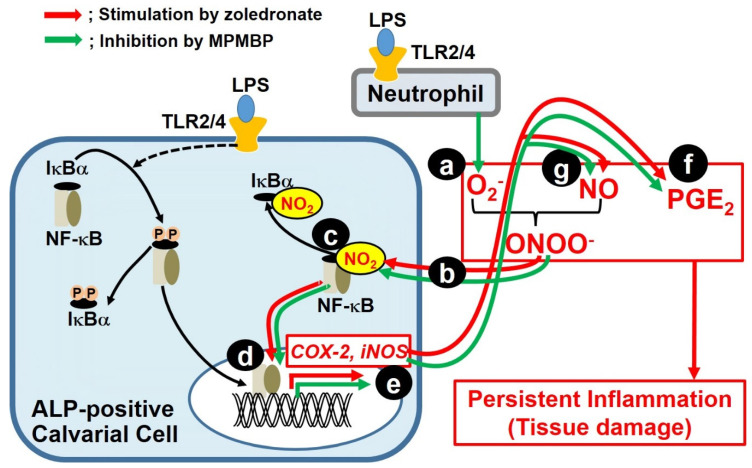
Proposed mechanism underlying the differential effects of N-containing zoledronate and non-N-containing MPMBP, on the inflammatory responses. The hypothesis is based on the experimental findings in the present study and published reports. We hypothesized that persistent inflammation leading to tissue damage might be caused by the signaling pathway as follows: (a) the LPS-stimulated production of O_2_^−^ by neutrophils; (b) peroxynitrite, which was produced from superoxide anions and NO, freely passes through the cell membrane; and (c) nitration of the tyrosine residue of IκB, which was reported to be a candidate for nitration target [33,35,36]; (d) translocation of activated NF-κB into the nucleus and thereby, (e) upregulation of *COX-2* and *iNOS* mRNA expression, which, in turn, increases the production of (f) PGE_2_ and (g) NO. This pathway from (a) to (g) is a vicious cycle that leads to persistent inflammation.

Calvaria used in this study contained several types of cells, including bone cells (osteoblasts, osteoclasts, and osteocytes), neutrophils, macrophages, fibroblasts, and osteoblast progenitor cells in the periosteum and bone marrow. Although the cell type which contributes to LPS-induced PGE_2_ production in the cultured calvaria is thought not to be single, since PGE_2_ was reported to be produced mainly by osteoblasts in bone [26], in response to various stimuli [27], our current results indicate that osteoblasts are considered to be one of cell types that produce PGE_2_ through NF-κB activation in calvaria. Meanwhile, activated macrophages (M1 polarized) are also known to play a critical role in inflammatory diseases, through the excessive production of inflammatory mediators, including PGE_2_ and NO, as well as pro-inflammatory cytokines [28,29,30], in which a transcription factor NF-κB is involved. Additionally, our recent investigation showed that MPMBP significantly inhibited Toll-like receptor 2 agonist-induced NF-κB/p65 activation in the macrophage-like cell line, J774.1 [31]. This evidence suggests that the activated macrophage might be a candidate for inflammation-responsive cell types in the calvaria and that MPMBP would be able to suppress inflammatory responses by acting against macrophages in the calvaria. When cells are stimulated with various pathogens, IκB is degraded post-phosphorylation, which leads to the activation of the oxidative stress-responsive transcription factor NF-κB. On the other hand, there is an alternate pathway, in which the tyrosine residue of IκB is nitrated by ONOO^−^, the extremely reactive molecule produced from NO and O_2_^−^ [24,32]. This can cause NF-κB activation for a longer period than that observed for IκB phosphorylation [33], without the degradation of IκB [34]. The present study showed that zoledronate strongly enhanced the production of PGE_2_ and NO; this might be attributable to the activation of NF-κB, which is concomitant with the increase in the cytoplasmic level of nitrotyrosine (Figure 6Ed). This finding indicates that the tyrosine nitration of IκB is thought to be a possible mechanism for NF-κB activation by zoledronate. Although previous reports show that IκB is one of the candidates to be nitrated on its tyrosine [33,35,36], further investigations using immunoprecipitation and/or immunohistochemistry analyses in calvaria would enable us to identify the protein to which the tyrosine is nitrated by ONOO^−^. Taken together, these findings raise the possibility that once calvaria is stimulated with LPS, neutrophils generate a great amount of O_2_^−^, which can produce cytotoxic ONOO^−^, by reacting with the NO produced by osteoblasts and/or activated macrophages. As a consequence, ONOO^−^ activates NF-κB, which results in the upregulation of inflammation-related genes, including *COX-2* and *iNOS* in the osteoblasts and/or macrophages (Scheme 1).

Szabo et al. showed that the sites of peroxynitrite formation are spatially associated with sources of O_2_^−^, reflecting its much shorter half-life and lower biomembrane permeability, owing to its negative charge, as compared to that of NO [37]. On the other hand, previous reports have shown that ONOO^−^ was a potent nitrating agent with a short half-life (<1 s) [38], and both motor neuron apoptosis and nitrotyrosine were prevented either by the inhibition of NO synthesis or O_2_^−^ scavengers [39]. This indicates the significance of ONOO^−^ as a biological effector molecule that can diffuse freely and rapidly across phospholipid membrane bilayers and react with target substrates [40]; this might support the hypothesis shown in Scheme 1. Furthermore, zoledronate accelerates this vicious cycle, which leads to uncontrolled persistent inflammation, while MPMBP may prevent tyrosine nitration via its O_2_^−^ scavenging activity. Although the inhibitory effect of MPMBP on O_2_^−^ production was not assessed in the cultured calvaria in this study, it is likely that MPMBP acts as an O_2_^−^ scavenger in the calvaria, as otherwise a large amount of O_2_^−^ would be produced by neutrophils in the presence of LPS. Since it was reported that the methylthio-residue could be s-demethylated by endogenous enzymes [41,42], it was possible that the thiol group (-SH) produced from the methylthio moiety of MPMBP functioned as an antioxidant, in a manner similar to that of SH-containing pyrrolidine dithiocarbamate (PDTC), a specific NF-κB inhibitor that was shown to inhibit the LPS-induced expression of *COX-2* [43] and *iNOS* [44] mRNA. In contrast to antioxidant activity of MPMBP (Figure 6C, left panel), none of the NBPs exhibited an O_2_^−^ scavenging activity (Figure 6C, right panel).

In addition, the association between long-term or high-dose NBPs and decreased plasma levels of antioxidants such as coenzyme Q10 (CoQ10), which serves as an endogenous antioxidant in every cell, has been demonstrated in postmenopausal women [45]. Furthermore, previous reports have demonstrated that gingival tissues from patients with periodontal disease showed a deficiency in CoQ10 [46,47], which supports this finding. Since the inhibition of FPP synthase by NBPs results in decreased levels of production of CoQ10 due to the shortage of precursor FPP [48,49], and no redistribution between organs occurs via circulation [50], it is likely that the reduced levels of antioxidants, such as CoQ10, could cause NBP-associated persistent inflammation, which leads to BRONJ.

Since BRONJ was first reported as a rare but serious adverse effect of BP by Marx in 2003 [51], the clinical use of BPs has been hampered at times, in spite of its beneficial effects on the risk of fracture in osteoporosis patients. Although the precise mechanisms for the onset of BRONJ remain unclear, accumulating evidence indicates that BRONJ is associated with invasive dental procedures, including teeth extraction, dental implant placement, periapical surgery, and periodontal surgery [11], in which a great number of neutrophils can be observed. This would generate a large amount of O_2_^−^, thereby leading to the production of ONOO^−^, which causes tissue damage. LPS, a pathogen used in the present study, is a major anaerobic bacterial component that causes inflammation in oral tissues, which are prone to be exposed to microorganisms, and a great number of pathological studies have suggested that BRONJ is an inflammation-associated process [52,53]. Thus, the stimulatory effects of NBPs on the inflammatory responses in alveolar bone and surrounding tissues might be related to the onset and development of BRONJ. Findings regarding the stimulatory effects of zoledronate on inflammation in this study, which are depicted in Scheme 1, are likely to explain the pathogenesis of BRONJ. The anti-inflammatory action of MPMBP was attributable to its potent antioxidant activity, as evidenced by the O_2_^−^ scavenging action in both human PMN [13] and the xanthine-xanthine oxidase system (Figure 6C). The consequent suppression of ONOO^−^ production might result in a lower incidence of BRONJ, as compared with that of patients treated with NBPs. Further studies by using BRONJ animal model would be one of the strategies to examine the relevance of our hypothesis which is depicted in Scheme 1. In addition to BRONJ, the acute-phase response, another adverse effect of NBPs featured by influenza-like symptoms, has been shown to be attributed to the release of proinflammatory cytokines [10].

In the present study, we selected the dose of BPs in order to obtain comparable anti-resorptive activities among different BPs (i.e., between 0.1–62.5 μM and 1–125 μM for NBPs and non-NBPs, respectively). This would enable us to obtain the relevant estimates of the balance between their efficacy as anti-resorption agents and the safety of therapeutics. It is well known that BPs have extremely low oral bioavailability (at a range of 0.6 to 1.5% of the administered dose [54,55]) due to their negative charge. On the other hand, all BPs have a strong affinity for hydroxyapatite in bone tissue because of common P-C-P chemical structure; therefore, they can be retained in bone for a long time which enables them to act primarily on the bone. Rodan described the cellular and molecular processes in periodontitis, in which the accumulated bacteria caused the destruction of the periodontium and therefore of possible therapeutic targets, which were likely to be similar to those used in rheumatoid arthritis patients [56], an inflammatory bone disease. Furthermore, Sandhu showed that nitrotyrosine-containing proteins were found in essentially all synovia from rheumatoid arthritis and osteoarthritis patients [57]. Taken together with the previous findings, which demonstrated that MPMBP treatment prevented against the inflammation-related bone loss in animal models [14,15,16,18], it is possible that MPMBP can act as an effective anti-resorptive agent with less adverse effects, including BRONJ and acute phase response, as compared to most currently available NBPs.

Another problem associated with BRONJ is that fixed treatment strategies remain unknown at present [11]. Recently, Hokugo et al. showed a unique strategy to reverse the adverse effect of zoledronate in mice. It was observed that the local administration of a “rescue” BP other than zoledronate dramatically reduced the development of osteonecrosis symptoms [58]. Since the course of BRONJ is progressive and greatly influenced by exposure to surrounding microorganisms in the oral cavity, which leads to uncontrolled inflammation, MPMBP may be a useful compound as a “rescue” BP, due to the most potent antioxidant and anti-inflammatory activities among currently available BPs.

## 5. Conclusions

All the examined NBPs (pamidronate, alendronate, incadronate, risedronate, zoledronate) stimulated the LPS-induced production of PGE_2_ and NO, by upregulating the expression of *COX-2* and *iNOS* mRNA, whereas non-NBPs (etidronate, clodronate, tiludronate, MPMBP) suppressed production of PGE_2_ and NO, by downregulating gene expression. Among non-NBPs, MPMBP exhibited the most potent anti-inflammatory activity via the inhibition of NF-κB/p65 nuclear translocation and tyrosine nitration of cytoplasmic proteins, by acting as an O_2_^−^ scavenger. These findings indicate that MPMBP can act as an efficacious anti-resorptive agent exhibiting less adverse effects in inflammatory bone diseases, including periodontitis and rheumatoid arthritis.

## 6. Patents

The content of this manuscript partially correlates to that of our MPMBP patents (Japanese patent: 5655179 and European patent: 2340841).

## Figures and Tables

**Table 1:** Specific primer sequences.

Genes	Primer Sequences (5′-3′)		Product Size (bp)	Accession Number
	Forward	Reverse		
*COX-1*	acc tgc ccc tat gtt tcc tt	cct tcc aag tgc tgg gat ta	421	NM_008969.4
*COX-2*	aag aca gat cat aag cga gga c	gcc agt gat aga gtg tgt tga a	532	NM_011198.4
*iNOS*	ctg ttc tca gcc caa caa tac a	gct ggt agg ttc ctg ttg ttt c	423	NM_010927.4
*eNOS*	agg aag gag tct agc aac aca gac	gtg agt cag ccc tgg tag taa ttg	369	NM_008713.4
*nNOS*	gct gtc cta tac agc ttc cag agt	caa agt tgt ctc tga ggt ctg gtc	700	NM_008712.3
*Actb*	tgt tac caa ctg gga cga ca	atg cca cag gat tcc ata cc	597	NM_007393.5

## Supplementary Materials

The following are available online at https://www.mdpi.com/2076-3921/9/6/503/s1, Figure S1: Representative confocal microscopy images of fluorescent-stained neonatal mouse calvaria.

## References

[1] Russell R.G. (2011). Bisphosphonates: The first 40 years. Bone.

[2] Rogers M.J., Watts D.J., Russell R.G. (1997). Overview of bisphosphonates. Cancer.

[3] Fleisch H. (2000). Bisphosphonates in Bone Disease. From the Laboratory to the Patient.

[4] van Beek E.R., Cohen L.H., Leroy I.M., Ebetino F.M., Löwik C.W.G., Papapoulos S.E. (2003). Differentiating the mechanisms of antiresorptive action of nitrogen containing bisphosphonates. Bone.

[5] Rogers M.J., Gordon S., Benford H.L., Coxon F.P., Luckman S.P., Monkkonen J., Frith J.C. (2000). Cellular and molecular mechanisms of action of bisphosphonates. Cancer.

[6] Khosla S., Burr D., Cauley J., Dempster D.W., Ebeling P.R., Felsenberg D., Gagel R.F., Gilsanz V., Guise T., Koka S. (2007). Bisphosphonate-associated osteonecrosis of the jaw: Report of a task force of the American society for bone and mineral research. J. Bone Miner. Res..

[7] Durie B.G.M., Katz M., Crowley J. (2005). Osteonecrosis of the jaw and bisphosphonates. N. Engl. J. Med..

[8] Bamias A., Kastritis E., Bamia C., Moulopoulos L.A., Melakopoulos I., Bozas G., Koutsoukou V., Gika D., Anagnostopoulos A., Papadimitriou C. (2006). Osteonecrosis of the jaw in cancer after treatment with bisphosphonates: Incidence and risk factors. J. Clin. Oncol..

[9] Hoff A.O., Toth B.B., Altundag K., Johnson M.M., Warneke C.L., Hu M., Nooka A., Sayegh G., Guarneri V., Desrouleaux K. (2008). Frequency and risk factors associated with osteonecrosis of the jaw in cancer patients treated with intravenous bisphosphonates. J. Bone Miner. Res..

[10] Hewitt R.E., Lissina A., Green A.E., Slay E.S., Price D.A., Sewell A.K. (2005). The bisphosphonate acute phase response: Rapid and copious production of proinflammatory cytokines by peripheral blood γφ T cells in response to aminobisphosphonates is inhibited by statins. Clin. Exp. Immunol..

[11] Kumar V., Shahi A.K. (2014). Nitrogen containing bisphosphonates associated osteonecrosis of the jaws: A review for past 10 year literature. Dent. Res. J..

[12] Takizawa A., Chiba M., Ota T., Yasuda M., Suzuki K., Kanemitsu T., Itoh T., Shinoda H., Igarashi K. (2016). The novel bisphosphonate disodium dihydrogen-4-[(methylthio) phenylthio] methane bisphosphonate increases bone mass in post-ovariectomy rats. J. Pharmacol. Sci..

[13] Tanahashi M., Funaba Y., Tateishi A., Kawabe N., Nakadate-Matsushita T. (1998). TRK-530 inhibits accumulation of superoxide anions derived from human polymorphonuclear leukocytes and bone resorption induced by activated osteoclasts. Pharmacology.

[14] Takaoka Y., Nagai H., Mori H., Tanahashi M. (1997). The effect of TRK-530 on experimental arthritis in mice. Biol. Pharm. Bull..

[15] Tanahashi M., Koike J., Kawabe N., Nakadate-Matsushita T. (1998). Inhibitory effect of TRK-530 on inflammatory cytokines in bone marrow of rats with adjuvant arthritis. Pharmacology.

[16] Tanahashi M., Funaba Y., Itoh M., Kawabe N., Nakadate-Matsushita T. (1998). Inhibitory effects of TRK-530 on rat adjuvant arthritis. Pharmacology.

[17] Attia A.M.M., Ibrahim F.A.A., Abd El-Latif N.A., Aziz S.W., Elwan A.M., Aziz A., Elgendy A., Elgengehy F.T. (2015). Role of reactive nitrogen species and antioxidant defense systems in the pathogenesis of rheumatoid arthritis. Wulfenia.

[18] Shinoda H., Takeyama S., Suzuki K., Murakami S., Yamada S. (2008). Pharmacological topics of bone metabolism: A novel bisphosphonate for the treatment of periodontitis. J. Pharmacol. Sci..

[19] Suzuki K., Takeshita F., Yamamoto K., Yamada S., Shinoda H., Ochiya T. In vivo imaging of osteoclast precursor recruitment to the inflammatory site where extensive bone destruction occurs. Proceedings of the ASBMR 2010 Annual Meeting.

[20] Nathan C. (2002). Points of control in inflammation. Nature.

[21] Lee K.W., Jeong S.Y., Park H.J., Jung H.J., Cho Y.W., Yun K., Lee K.T. (2008). Inhibition of LPS-induced NO and PGE2 production by asiatic acid via NF-kappa B inactivation in RAW 264.7 macrophages: Possible involvement of the IKK and MAPK pathways. Int. Immunopharmacol..

[22] Suzuki K., Takeyama S., Kikuchi T., Yamada S., Sodek J., Shinoda H. (2005). Osteoclast responses to lipopolysaccharide, parathyroid hormone and bisphosphonates in neonatal murine calvaria analyzed by laser scanning confocal microscopy. J. Histochem. Cytochem..

[23] Pikarsky E., Porat R.M., Stein I., Abramovitch R., Amit S., Kasem S., Gutkovich-Pyest E., Urieli-Shoval S., Galun E., Ben-Neriah Y. (2004). NF-kappaB functions as a tumour promoter in inflammation-associated cancer. Nature.

[24] Hogg N., Darley-Usmar V.M., Wilson M.T., Moncada S. (1992). Production of hydroxyl radicals from the simultaneous generation of superoxide and nitric oxide. Biochem. J..

[25] Hosfield D.J., Zhang Y., Dougan D.R., Broun A., Tari L.W., Swanson R.V., Finn J. (2003). Structural basis for bisphosphonate-mediated inhibition of isoprenoid biosynthesis. J. Biol. Chem..

[26] Miyaura C., Inada M., Matsumoto C., Ohshiba T., Uozumi N., Shimizu T., Ito A. (2003). An essential role of cytosolic phospholipase A2alpha in prostaglandin E2-mediated bone resorption associated with inflammation. J. Exp. Med..

[27] Wadleigh D.J., Herschman H.R. (1999). Transcriptional regulation of the cyclooxygenase-2 gene by diverse ligands in murine osteoblasts. Biochem. Biophys. Res. Commun..

[28] Watson W.H., Zhao Y., Chawla R.K. (1999). *S*-adenosylmethionine attenuates the lipopolysaccharide-induced expression of the gene for the tumour necrosis factor-α. Biochem. J..

[29] Kubes P., McCafferty D.M. (2000). Nitric oxide and intestinal inflammation. Am. J. Med..

[30] Gu Q., Yang H., Shi Q. (2017). Macrophages and bone inflammation. J. Orthop. Translat..

[31] Tamai R., Suzuki K., Mashima I., Kiyoura Y. (2020). MPMBP down-regulates Toll-like receptor (TLR) 2 ligand-induced proinflammatory cytokine production by inhibiting NF-κB but not AP-1 activation. Int. Immunopharmacol..

[32] Ischiropoulos H. (1998). Biological tyrosine nitration: A pathophysiological function of nitric oxide and reactive oxygen species. Arch. Biochem. Biophys..

[33] Gochman E., Mahajna J., Reznick A.Z. (2011). NF-κB activation by peroxynitrite through IκBα-dependent phosphorylation versus nitration in colon cancer cells. Anticancer Res..

[34] Janssen-Heininger Y.M., Macara I., Mossman B.T. (1999). Cooperativity between oxidants and tumor necrosis factor in the activation of nuclear factor (NF)-kappaB: Requirement of Ras/mitogen-activated protein kinases in the activation of NF-kappaB by oxidants. Am. J. Respir. Cell Mol. Biol..

[35] Yakovlev V.A., Barani I.J., Rabender C.S., Black S.M., Leach J.K., Graves P.R., Kellogg G.E., Mikkelsen R.B. (2007). Tyrosine nitration of IκBα: A novel mechanism for NF-κB activation. Biochemistry.

[36] Nagaoka M., Maeda T., Chatani M., Handa K., Yamakawa T., Kiyohara S., Negishi-Koga T., Kato Y., Takami M., Niida S. (2019). A delphinidin-enriched maqui berry extract improves bone metabolism and protects against bone loss in osteopenic mouse models. Antioxidants.

[37] Szabó C., Módis K. (2010). Pathophysiological roles of peroxynitrite in circulatory shock. Shock.

[38] Pfeiffer S., Gorren A.C.F., Schmidt K., Werner E.R., Hansert B., Bohle D.S., Mayer B. (1997). Metabolic fate of peroxynitrite in aqueous solution: Reaction with nitric oxide and pH-dependent decomposition to nitrite and oxygen in a 2:1 stoichiometry. J. Biol. Chem..

[39] Estévez A.G., Spear N., Manuel S.M., Radi R., Henderson C.E., Barbeito L., Beckman J.S. (1998). Nitric oxide and superoxide contribute to motor neuron apoptosis induced by trophic factor deprivation. J. Neurosci..

[40] Marla S.S., Lee J., Groves J.T. (1997). Peroxynitrite rapidly permeates phospholipid membranes. Proc. Natl. Acad. Sci. USA.

[41] Mazel P., Henderson L.F., Axelrod J. (1964). S-demethylation by microsomal enzymes. J. Pharmacol. Exp. Ther..

[42] Larsen G.L., Bakke J.E., Feil V.J., Huwe J.K. (1988). In vitro metabolism of the methylthio group of 2-methylthiobenzothiazole by rat liver. Xenobiotica.

[43] Liu S.F., Ye X., Malik A.B. (1999). Inhibition of NF-κB activation by pyrrolidine dithiocarbamate prevents in vivo expression of proinflammatory genes. Circulation.

[44] Liu S.F., Ye X., Malik A.B. (1997). In vivo inhibition of nuclear factor-kappa B activation prevents inducible nitric oxide synthase expression and systemic hypotension in a rat model of septic shock. J. Immunol..

[45] Kalyan S., Huebbe P., Esatbeyoglu T., Niklowitz P., Côté H.C.F., Rimbach G., Kabelitz D. (2014). Nitrogen-bisphosphonate therapy is linked to compromised coenzyme Q10 and vitamin E status in postmenopausal women. J. Clin. Endocrinol. Metab..

[46] Littarru G.P., Nakamura R., Ho L., Folkers K., Kuzell W.C. (1971). Deficiency of coenzyme Q 10 in gingival tissue from patients with periodontal disease. Proc. Natl. Acad. Sci. USA.

[47] Prakash S., Sunitha J., Hans M. (2010). Role of coenzyme Q (10) as an antioxidant and bioenergizer in periodontal diseases. Indian J. Pharmacol..

[48] Dallner G., Sindelar P.J. (2000). Regulation of ubiquinone metabolism. Free Radic. Biol. Med..

[49] Nawarskas J.J. (2005). HMG-CoA Reductase inhibitors and coenzyme Q10. Cardiol. Rev..

[50] Elmberger P.G., Kalen A., Appelkvist E.L., Dallner G. (1987). In vitro and in vivo synthesis of dolichol and other main mevalonate products in various organs of the rat. Eur. J. Biochem..

[51] Marx R.E. (2003). Pamidronate (Aredia) and zoledronate (Zometa) induced avascular necrosis of the jaws: A growing epidemic. J. Oral Maxillofac. Surg..

[52] Lesclous P., Abi N.S., Carrel J.P., Baroukh B., Lombardi T., Willi J.P., Rizzoli R., Saffar J.L., Samson J. (2009). Bisphosphonate-associated osteonecrosis of the jaw: A key role of inflammation?. Bone.

[53] Iannitti T., Rosini S., Lodi D., Frediani B., Rottigni V., Palmieri B. (2012). Bisphosphonates: Focus on inflammation and bone loss. Am. J. Ther..

[54] Khosla S., Bilezikian J.P., Dempster D.W., Lewiecki M.E., Miller P.D., Neer R.M., Recker R.R., Shane E., Shoback D., Potts J.T. (2012). Benefits and risks of bisphosphonate therapy for osteoporosis. J. Clin. Endocrinol. Metab..

[55] Cremers S., Papapoulos S. (2011). Pharmacology of bisphosphonates. Bone.

[56] Rodan G.A., Martin T.J. (2000). Therapeutic approaches to bone diseases. Science.

[57] Sandhu J.K., Robertson S., Birnboim H.C., Goldstein R. (2003). Distribution of protein nitrotyrosine in synovial tissues of patients with rheumatoid arthritis and osteoarthritis. J. Rheumatol..

[58] Hokugo A., Kanayama K., Sun S., Morinaga K., Sun Y., Wu Q., Sasaki H., Okawa H., Evans C., Ebetino F.H. (2019). Rescue bisphosphonate treatment of alveolar bone improves extraction socket healing and reduces osteonecrosis in zoledronate-treated mice. Bone.

