# Supplementary material for: Structure-Dependent Effects of Bisphosphonates on Inflammatory Responses in Cultured Neonatal Mouse Calvaria
Source: Antioxidants (Basel). 2020 Jun 9;9(6):503. doi: 10.3390/antiox9060503 (PMC7346192; doi:10.3390/antiox9060503)
Supplement: Supplementary file 1 [file antioxidants-09-00503-s001.pdf]

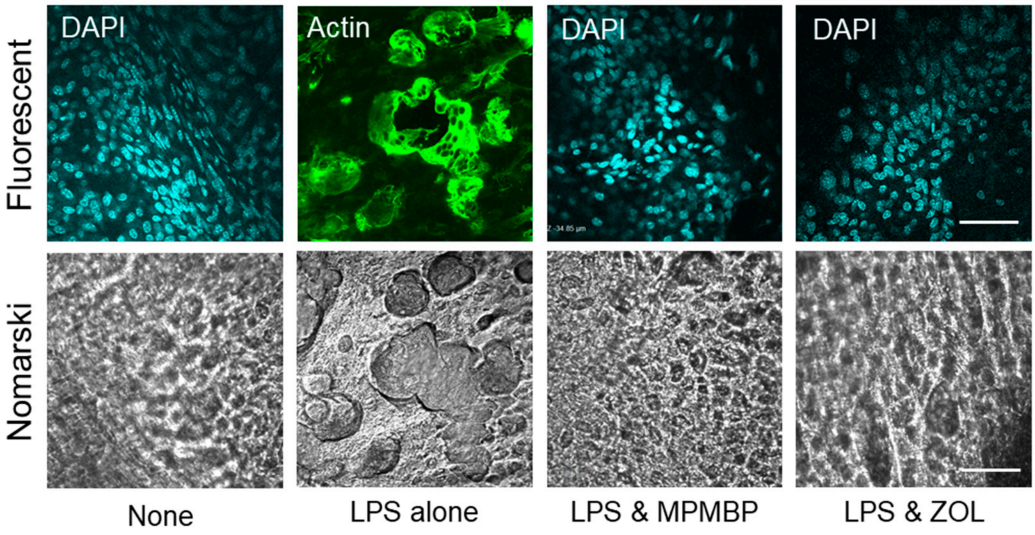

Figure S1: Representative confocal microscopy images of fluorescent-stained neonatal mouse calvaria. Parietal bones were incubated with the vehicle, 10 µg/mL LPS alone, LPS & 125 µM MPMBP or LPS & 25 µM zoledronate for 48 h. Scale bars = 50 µm.
